# *Toxoplasma* Chinese 1 Strain of WH3Δ*rop16_I/III_*/*gra15_II_* Genetic Background Contributes to Abnormal Pregnant Outcomes in Murine Model

**DOI:** 10.3389/fimmu.2018.01222

**Published:** 2018-06-01

**Authors:** Cong Wang, Weisheng Cheng, Qian Yu, Tian Xing, Shoubin Chen, Lei Liu, Li Yu, Jian Du, Qingli Luo, Jilong Shen, Yuanhong Xu

**Affiliations:** ^1^Department of Pathogen Biology, Provincial Laboratories of Pathogen Biology and Zoonoses, School of Basic Medicine, Anhui Medical University, Hefei, China; ^2^Department of Medical Genetics, Zhongshan School of Medicine, Sun Yat-sen University, The Key Laboratory of Tropical Disease Control, Ministry of Education, Guangzhou, China; ^3^Department of Clinical Laboratory, The First Affiliated Hospital of Anhui Medical University, Hefei, China

**Keywords:** *Toxoplasma gondii*, dense granule protein GRA15, rhoptry protein ROP16, CRISPR/Cas9, adverse pregnant outcome

## Abstract

*Toxoplasma gondii* infection evokes a strong Th1-type response with interleukin (IL)-12 and interferon (IFN)-γ secretion. Recent studies suggest that the infection of pregnant mice with *T. gondii* may lead to adverse pregnancy results caused by subversion of physiological immune tolerance at maternofetal interface rather than direct invasion of the parasite. Genotype-associated dense granule protein GRA15_II_ tends to induce classically activated macrophage (M1) differentiation and subsequently activating NK, Th1, and Th17 cells whereas rhoptry protein ROP16_I/III_ drives macrophages to alternatively activated macrophage (M2) polarization and elicits Th2 immune response. Unlike the archetypal strains of types I, II, and III, type Chinese 1 strains possess both GRA15_II_ and ROP16_I/III_, suggesting a distinct pathogenesis of *Toxoplasma*-involved adverse pregnancies. We constructed *T. gondii* type Chinese 1 strain of WH3Δ*rop16* based on CRISPR/Cas9 technology to explore the ROP16_I/III_-deficient/GRA15_II_-dominant parasites in induction of trophoblast apoptosis *in vitro* and abnormal pregnant outcomes of mice *in vivo*. Our study showed that *Toxoplasma* WH3Δ*rop16* remarkably induced apoptosis of trophoblasts. C57BL/6 pregnant mice injected with the tachyzoites of WH3Δ*rop16* presented increased absorptivity of fetuses in comparison with the mice infected with WH3 wild type (WH3 WT) parasites although no remarkable difference of virulence to mice was seen between the two strains. Additionally, the mice inoculated with WH3Δ*rop16* tachyzoites exhibited a notable expression of both IL-17A and IFN-γ, while the percentage of CD4^+^CD25^+^FoxP3 [T regulatory cells (Tregs)] were diminished in splenocytes and placenta tissues compared to those infected with WH3 WT parasites. Accordingly, expressions of IL-4, IL-10, and transforming growth factor beta 1, the pivotal cytokines of Th2 and Tregs response, were significantly dampened whereas IFN-γ and IL-12 expressions were upregulated in WH3Δ*rop16*-infected mice, which gave rise to more prominent outcomes of abnormal pregnancies. Our results indicated that the WH3Δ*rop16* parasites with *gra15_II_* background of *T. gondii* type Chinese 1 strains may cause miscarriage and stillbirth due to subversion of the maternal immune tolerance and system immunity of the animals and the GRA15_II_ effector contributes to the process of adverse pregnant consequences.

## Introduction

During normal pregnancy, allogeneic fetal cells invading the extraembryonic trophoblasts do not impair gestation by establishing tolerance at the maternal–fetal interface (1, 2). The physiological balance of Th1/Th2 is believed to play a crucial part in maintenance of normal pregnancy of mammals including humans (3). Previous studies showed that apoptosis is a normal physiological process of trophoblasts throughout gestation and is essential to normal placental development and fetal growth (4). Thus any unfavorable impact of immunological factors on the maternal–fetal interface and/or apoptosis of trophoblasts may lead to abnormal pregnancy outcomes.

It has been elucidated that T regulatory cells (Tregs) promote immune tolerance and successful pregnancy by secreting interleukin (IL)-10 and transforming growth factor beta 1 (TGF-β1) and dampen interferon (IFN)-γ and other inflammatory cytokines to maintain the normal development of embryos (5–8). The Tregs cells have been found to be insufficient in patients who experienced recurrent spontaneous abortions (9), while excessive secretion of IFN-γ and other Th1 type cytokines may give rise to adverse pregnancies (10). Therefore, Th2-dominant response in system immunity and at maternal–fetal interface is the prerequisite for normal pregnancy (3, 11–14).

*Toxoplasma gondii* is an extensive intracellular protozoan parasite that is capable of affecting almost all vertebrate animals including humans and leading to reproductive failure in its hosts (15, 16). Primary infection with *Toxoplasma* during pregnancy, particularly in the first trimester, may cause stillbirths, miscarriages, or fetal abnormalities (17). The apoptosis of trophoblasts might be induced by many stimuli. For example, apoptotic process of trophoblasts is notably increased in the case of spontaneous abortion when the cells were co-cultured with inflammatory macrophages that were infected with *T. gondii* (18). Glycosyl phosphatidylinositol on the membrane of *Toxoplasma* tachyzoites, similar to LPS of bacteria, could induce oxidative stress in several tissues, and it has been found to be a key molecule which is responsible for preterm labor in mice (19, 20). Despite the fact that *Toxoplasma* infection may cause abortion or congenital fetal infection *via* direct transmission of parasite to placentas and fetuses, some studies indicate that imbalance of immune tolerance at the maternal–fetal interface, rather than a direct action of the parasite, might be attributed to the abnormal pregnancies (21, 22), resulting in apoptosis of trophoblasts in the case of abortion with *T. gondii* infection. Further researches revealed that *Toxoplasma* infection in mice lead to adverse pregnant results with a mechanism of reduction of Tregs and elevation of Th17 cells (23).

*Toxoplasma gondii* invasion to the host cell is actually a series of parasite protein secretions. Secretory proteins of ROPs, GRAs, MICs, RONs, and other molecules are mainly generated by the organelles of dense granules, rhoptries, and micronemes. These parasite-derived polymorphic effectors are deeply involved in dictation of virulence and modulation of host signaling pathways (24–28). For instance, Jensen et al. found that ROP16_I/III_ (of types I and III strains) kinase phosphorylates Stat6/Stat3 and induces alternatively activated macrophages (M2) at early phase of infection that is associated with promoted IL-4 and IL-10 expressions and Th2-polarized response, while GRA15_II_ (of type II strains) activates NF-κB and elicits classically activated macrophages (M1) that is responsible for IL-12 generation and Th1-dominant immunity, subsequently activating NK and Th17 cells (19, 28–31), resulting in oxidative stress and further activation of the pro-apoptotic pathway (28, 32).

Studies reported that type Chinese 1 (ToxoDB #9) strains dominantly prevalent in China carry both GRA15_II_ and ROP16_I/III_ effectors that is different from the strains of archetypical types I, II, and III of *T. gondii* in Europe and North America (33, 34). The ROP16_I/III_ with GRA15_II_ background in Chinese 1 strains strongly suggests the distinct pathogenesis that differs from the strains of the other continents of the world. In the current study, we explored the impact of GRA15_II_ with ROP16_I/III_ deletion of Chinese 1 WH3 strain parasite on adverse pregnancy outcomes in murine model by making a *T. gondii* WH3Δ*rop16* strain based on CRISPR/Cas9 technology. We hypothesize that the Toxo-WH3Δ*rop16* strain with *gra15_II_* background, similar to type II strains of *Toxoplasma*, might subvert the immune tolerance at the maternal–fetal interface and the systemic immunity, leading to adverse pregnancy, which is attributed to the M1/Th1/Th17 biased response. Moreover, this study would be helpful for better understanding of the genotype-associated mechanism of abnormal pregnancy caused by *Toxoplasma* predominantly circulating in China.

## Materials and Methods

### Reagents

The following reagents were used in the experiment: Q5 mutagenesis kit (New England Biolabs, Ipswich, MA, USA) and ClonExpress MultiS One Step Cloning Kit (Vazyme Biotech, Nanjing, China). Phusion High-Fidelity PCR Kit (Thermo fisher, USA) was used for the PCR amplification. Percoll (GE Healthcare Life Sciences), pyrimethamine, penicillin, streptomycin, phorbol 12-myristate 13-acetate, ionomycin, HEPES, collagenase IV, and the Giemsa staining kit, were obtained from Sigma (St. Louis, MO, USA). Dulbecco’s modified Eagle’s medium, fetal bovine serum (FBS), and Roswell Park Memorial Institute 1640 medium (RPMI 1640) and D-Hanks solution were purchased from Wisent (Montreal, QC, Canada). Brefeldin A, FITC-labeled anti-mouse CD4, PE-labeled anti-mouse IL-17A, APC-labeled anti-mouse IL-4, Percp-cy5.5-labeled anti-mouse IFN-γ, APC-labeled anti-mouse CD25, PE-labeled anti-mouse Foxp3, FITC Annexin V Apoptosis Detection Kit, and Cytofix/Cytoperm kit were provided by BD Biosciences (New York, BD, USA). The nitrite was detected *via* Greiss Reagent System provided by Promega Biotech Company (Madison, WI, USA). The tumor necrosis factor (TNF-α), IL-17A enzyme-linked immunosorbent assay (ELISA) kit, and 1 × Fix/Perm buffer were purchased from eBioscience (San Diego CA, USA). IFN-γ, IL-12, IL-10, and TGF-β1 ELISA kit was obtained from R&D Systems (Minneapolis, MN, USA). The mouse trophoblasts (primary cells originated from C57BL/6 mice) were purchased from Wuhan Biofavor Biotechnology Company.

### Propagation of *T. gondii*

Tachyzoites of *T. gondii* WH3 strain (type Chinese 1) were harvested from the continuous cell cultures in human foreskin fibroblasts (HFF, ATCC^®^ SCRC-1041™) grown with Dulbecco’s modified Eagle medium (DMEM) supplemented with 10% FBS, 100 µg/ml streptomycin, and 100 U/ml penicillin.

### Generation of ROP16_I/III_-Deficient Strain of Type Chinese 1

All the primers and plasmids used in the study are shown in Table 1. UPRT-targeting guide RNA (gRNA) in pSAG1:CAS9-U6:sgUPRT (Addgene plasmid #54467) was replaced with ROP16_I/III_ targeting gRNA using Q5 mutagenesis kit. To make the homologous templates for gene replacements, homologous arms of ROP16_I/III_ were amplified from the genomic DNA of type Chinese 1 WH3 WT strain as described in previous study (35). Subsequently, the homologous arms of ROP16 along with the selectable markers DHFR*-Ts were cloned into pUC19 using ClonExpress MultiS One Step Cloning Kit. The pSAG1:Cas9-U6:sgROP16_I/III_ and donor DNA fragments were electroporated into *T. gondii* WH3 WT strain. After transfection, tachyzoites were immediately transferred into HFF cells. After culture for 48 h, parasites were selected with pyrimethamine for DHFR-TS. A single clone through limiting dilution in 96-well plate were seeded with HFF host cells and detected by PCR and Western blotting. Diagnostic PCRs (PCR1, PCR2, and PCR3) were used to screen individual clone. PCR reactions were performed using Taq DNA polymerase in a 25-µl reaction mixture containing 1 µl genomic DNA extracted from a single clone as templates. Subsequently, the products were examined by agarose gel electrophoresis. Rabbit polyclonal antibody against ROP18 and antibody against ROP16 was prepared in the laboratory. The invasion of WH3 WT or WH3Δ*rop16* strain in HFF cells were visualized after 48 h by Giemsa staining and the average number of tachyzoites per parasitophorous vacuole (PV) was obtained by counting 50 PVs in triplicate experiments. C57BL/6 mice were infected with 1 × 10^3^ tachyzoites of WH3 WT or WH3Δ*rop16*, respectively. The animals were monitored daily for manifestations following infection and the survival rate was recorded.

**Table 1:** Primers used in this study.

Primers	Sequence	Used for
5′-ROP16-guide RNA (gRNA)	GTGGCAGCGCGTTTTAGAGCTAGAAATAGCAAG	Q5 mutagenesis changing the gRNA in pSAG1:CAS9-U6:sgUPRT to gRNA-ROP16
3′-ROP16-gRNA	CGGTGCGTCCAACTTGACATCCCCATTTAC	

UpROP16 F	AAAACGACGGCCAGTGAATTCAGTTTGAATCTCTGGGTAGAACAGC	To produce UpROP16 PCR product for making pROP16:DHFR-I
UpROP16 R	GGGGGTGAAAATCGAATGACACTGCCCCTGAGTCGAGCCAC	

DHFR-TS F	TGTCATTCGATTTTCACCCCC	To produce DHFR PCR product for making pROP16:DHFR-I
DHFR-TS R	TCCTCCGCTCCTCTCTAGCAGGATCGATCCCCCCGGGCTGC	

DnROP16 F	GCAGCCCGGGGGGATCGATCCTGCTAGAGAGGAGCGGAGGA	To produce DnROP16 PCR product for making pROP16:DHFR-I
DnROP16 R	GACCATGATTACGCCAAGCTTGCTCCGCAGTCTCTGTAAGT	

5′-UpROP16	TGTGATGCTGAGTCTTGCGAGT	PCR1
5′-InDHFR	TACCAGTCATGGACGAGATCG	

3′-InDHFR	ACACGCATGTCTACACGAACC	PCR2
3′-DnROP16	TGGTGGTTGCGACTGGACTA	

5′-InROP16	ATGAAAGTGACCACGAAAGG	PCR3
3′-InROP16	CTACATCCGATGTGAAGAAAGTT	

### Cells and Co-Culture System

Mouse peritoneal macrophages were acquired by washing the peritoneal cavity three times with ice-cold D-Hanks solution. Mouse peritoneal macrophages were cultured and suspension cells were washed off 24 h later. The transwell culture plates (Corning, Corning, NY, USA), were used to establish a co-culture system. Generally, the pore size of the bottom of the inserts is 0.4 µm, which allowed the passage of only small and soluble particles rather than cells and tachyzoites. Control macrophages (1 × 10^6^) and WH3 WT (1 × 10^6^) or WH3Δ*rop16* (1 × 10^6^) tachyzoites infected macrophages were added to the upper well separately. The C57BL/6 primary placental trophoblasts (1 × 10^6^) were co-cultured in the lower wells in 5% CO_2_ at 37°C for 24 h. The co-culture system was kept in 1 ml of RPMI 1640 culture medium supplemented with 10% FBS and 1% penicillin/streptomycin. After co-culturing for 24 h, the macrophages were harvested for total RNA and culture supernatants were collected to analyze NO and TNF-α. The placental trophoblasts were collected for detection of apoptosis by flow cytometry (FCM). The apoptosis rates of trophoblasts cells were calculated as the sum of early and late apoptosis rates. The trophoblasts cells washed twice with cold PBS, and resuspended cells in 1 × binding buffer. The trophoblast cells were subjected to FITC annexin V and PI followed by gentle vortex, incubated for 15 min at 25°C in the dark and analyzed by FCM within 1 h.

### Establishment of Animal Pregnant Model

The 6- to 8-week-old female and 8- to 10-week-old male C57BL/6 mice were purchased from the Animal Center of Anhui Medical University (AMU) and had free access to sterilized water and food under standard conditions, and permitted to adapt for 1 week before the experiment. The mice were treated in compliance with the Chinese National Institute of Health Guide for the Care and Use of Laboratory Animals. All procedures were followed strictly according to the ethical standards formulated by Institutional Review Board of AMU Institute of Biomedicine AMU (permit No: AMU26-081108). After 1 week of adaptation to the new environment, females were housed overnight with males (2 females for 1 male). The presence of vaginal plugs on the second day was designated day 0 of gestation (GD 0). Briefly, all pregnant mice were divided into three equal groups randomly (WH3 WT group, WH3Δ*rop16* group, and control group) with five mice in each. On gestation GD8, pregnant mice were intraperitoneally injected with 4 × 10^2^ WH3 WT and WH3Δ*rop16* tachyzoites in 200 µl sterile PBS, and the control was exposed to only 200 µl sterile PBS at the same time. All mice were sacrificed with euthanasia on GD14, the uteruses were moved away and implantation and resorption sites were recorded. The resorption sites were defined by their small sizes, necrotic, and hemorrhagic appearance of placentas and embryos. The ratio of resorption sites to total implantation sites was calculated as the percentage of fetal loss.

The experiments that are related to using viable parasites and animal infections were performed in the AMU Biosecurity II Laboratory licensed by the local health administrative department.

### Preparation of Single Cell Suspension

The placentas, uterine tissues, and spleens of experimental mice were removed aseptically. The spleen tissues were placed in a sterile nylon mesh gently. The cells were harvested after mashing through sterile nylon gauze and lysed with erythrocyte lytic buffer and cultured in RPMI 1640 supplemented with 10% FBS. At the same time, the placentas and the uterus were placed in the dish and cut into pieces followed by washing thoroughly and the shredded tissue was moved to a 50 ml centrifuge tube. The tissues digested with 2.5 mg/ml collagenase IV, 10% FBS, and 10 mM HEPES sodium salt dissolved in RPMI 1640 and then incubated at 37°C for 30 min with gentle shaking. The suspension cells were filtered through sterile nylon mesh to take away undigested tissue. The mononuclear cells were collected by discontinuous 70%/40% Percoll (GE Healthcare Life Sciences) density gradient centrifugation.

### FCM Analysis of Lymphocytes

Lymphocytes from spleens, placentas, and uterine tissues were regulated to the appropriate cell number (1 × 10^6^/ml) and cultivated in 6-well plate in 2 ml of RPMI 1640 medium supplemented with 10% FBS. For intracellular cytokine staining, ionomycin (1 mg/ml) and PMA (20 ng/ml) were used to stimulate the cells for 5 h in the presence of brefeldin A (1 mg/ml). The cells were subjected to FITC-labeled anti-mouse CD4 for 30 min at 4°C to avoid the light and were washed twice, and fixed with the Cytofix/Cytoperm kit according to manufacturer’s instructions. The cells were incubated for 1 h at 4°C in the dark and were washed twice, and PE-labeled anti-mouse IL-17A, APC-labeled anti-mouse IL-4, and Percp-cy5.5-labeled anti-mouse IFN-γ were used for staining of intracellular cytokines, respectively. Tregs were marked by the FITC-labeled anti-mouse CD4 and APC-labeled anti-mouse CD25 for 30 min at 4°C in the dark and were washed twice. After surface staining, the cells were fixed and permeabilized in 1 × Fix/Perm buffer at 4°C for 1 h in the dark and washed twice, followed by PE-labeled anti-mouse Foxp3 staining at 4°C for 30 min in protection from light. After washing, the cells were analyzed on FCM.

### RNA Extraction and qRT-PCR

Total RNA from the co-cultured cells, spleens, and placentas were extracted using TRIzol (Invitrogen Life Technologies, Carlsbad, CA, USA) and transcribed into cDNA using Takara Kit (Takara, Japan) following the manufacturer’s instructions. The quantitative analysis was performed by testing expression of iNOS, TNF-α, IFN-γ, IL-12, IL-17A, IL-10, and TGF-β1 using SYBR Premix Ex Taq kit (Takara, Japan) by the Light Cycler 480. The glyceraldehyde-3-phosphate dehydrogenase (GAPDH) was used to normalize the results. The relative mRNA expression was counted with the comparative ΔCt method using the formula 2^−ΔΔCt^. The qRT-PCR was conducted in technical triplicates by using the sense and antisense primers listed in Table 2.

**Table 2:** The primers used for qRT-PCR.

Primers	Forward primer (5′–3′)	Reverse primer (5′–3′)
TNF-α	ACGGCATGGATCTCAAAGAC	GTGGGTGAGGAGCACGTAGT
iNOS	CACCTTGGAGTTCACCCAGT	ACCACTCGTACTTGGGATGC
IFN-γ	AGCAAGGCGAAAAAGGATGC	TCATTGAATGCTTGGCGCTG
IL-12	GATGTCACCTGCCCAACTG	TGGTTTGATGATGTCCCTGA
IL-10	GCTCCTAGAGCTGCGGACT	TGTTGTCCAGCTGGTCCTTT
TGF-β1	CTGGATACCAACTACTGCTTCAG	TTGGTTGTAGAGGGCAAGGACCT
IL-17A	TCTCTGATGCTGTTGCTGCT	CGTGGAACGGTTGAGGTAGT
GAPDH	CAACTTTGGCATTGTGGAAGG	ACACATTGGGGGTAGGAACAC

*GAPDH, glyceraldehyde-3-phosphate dehydrogenase; IFN, interferon; IL, interleukin; iNOS, inducible nitric synthase; TNF, tumor necrosis factor; TGF-β1, transforming growth factor beta 1*.

### Enzyme-Linked Immunosorbent Assay

The co-cultured supernatants were obtained for TNF-α detection by ELISA. To detect cytokines in splenocyte supernatants, we used the ionomycin (1 mg/ml) and PMA (20 ng/ml) to stimulate the splenocyte cells (2 × 10^6^) in 6-well plate in 2 ml/well of RPMI 1640 medium supplemented with 10% FBS for 5 h. Splenocyte supernatants were collected and subjected to examination for IFN-γ, IL-12, IL-17A, IL-10, and TGF-β1 by ELISA. Accurately weighed placenta tissues were homogenized with a ratio of 1 mg of placental tissues to 10 µl of PBS and centrifuged at 12,000 *g* at 4°C for 30 min. The supernatants of the tissue lysates were gathered and equal volume of the supernatants was added to each well. The cytokines of IFN-γ, IL-12, IL-17A, IL-10, and TGF-β1 were detected by ELISA according to the manufacturer’s protocols. Three duplicate wells were set up for each group. The absorbance was measured at 450 nm on the ELISA plate reader (Biotek, Winooski, VT, USA).

### Nitrite Detection

The co-cultured supernatant was collected for examination of nitrite oxide (NO). It was detected using the Griess Reagent system as previously described (36) following the manufacturer’s instruction. The absorbance was measured at 550 nm on an ELISA plate reader.

### Statistical Analysis

The experimental data acquired were subjected to statistical analyses using one-way ANOVA and paired *t*-test after precheck of the data for homogeneity of variances. Statistical significance was defined by using GraphPad Prism Software, and *P* < 0.05 was regarded as significant. The results were presented as mean ± SD, which gives a summary of the data from at least three times experiments.

## Results

### The WH3Δ*rop16* Strain of *T. gondii* Type Chinese 1 Was Constructed Using CRISPR/Cas9 Technology

**Figure 1:**
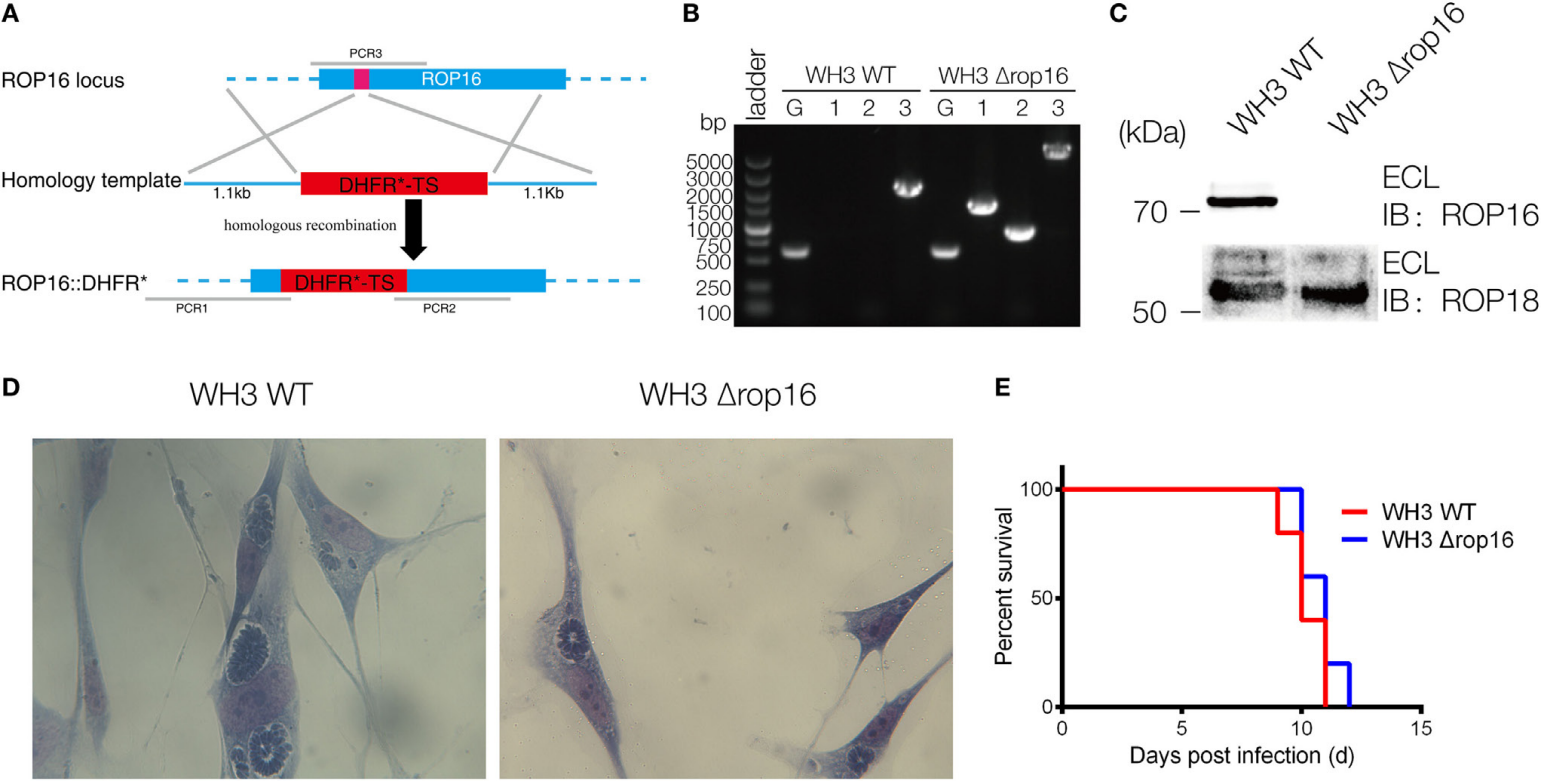
Construction of the WH3Δ*rop16* strain of *Toxoplasma gondii* type Chinese 1 by using CRISPR-Cas9 technology. **(A)** Schematic of CRISPR/CAS9 strategy for inactivation of *rop16* by inserting pyrimethamine-resistant DHFR (DHFR*) and PCR identification of single clone. **(B)** PCR identification of WH3Δ*rop16 T. gondii* strain. G represents the GRA1 promoter positive control, as shown in Figure 1A. 1: PCR1 fragment; 2: PCR2 fragment, 3: PCR3 fragment. PCR1 and PCR2 examined the integration of DHFR-TS* into corresponding genes, and PCR3 checked the deletion of ROP16 sequences. **(C)** Western blotting for detection of ROP16 expression in WH3 WT and WH3Δ*rop16*. Rabbit polyclonal antibody against ROP18 was used as the control. **(D)** The number of parasites per parasitophorous vacuole of WH3Δ*rop16* or WH3 WT. **(E)** Mouse survival after inoculation with *T. gondii* tachyzoites.

The CRISPR/Cas9 strategy was used to inactivate ROP16 by inserting pyrimethamine-resistant DHFR (DHFR*) followed by PCR identification of a single clone (Figure 1A). PCR identification proved that DHFR coding sequence was successfully inserted to the target position (Figure 1B). Western blotting analysis showed no expression of ROP16_I/III_ protein by WH3Δ*rop16* strain (Figure 1C). Giemsa staining results indicated that less number of parasites (13 tachyzoites vs 22 tachyzoites) per PV in WH3Δ*rop16* infected cells compared to WH3 WT-infected cells, respectively (*p* < 0.05) (Figure 1D). Virulence examination indicated that all animals infected by both strains died on day 12 post-infection (Figure 1E) and no difference of the virulence to mice was noted between WH3 WT and WH3Δ*rop16* strains.

### WH3Δ*rop16* Strain Infection Drove Macrophages to M1 and Lead to Trophoblast Apoptosis

**Figure 2:**
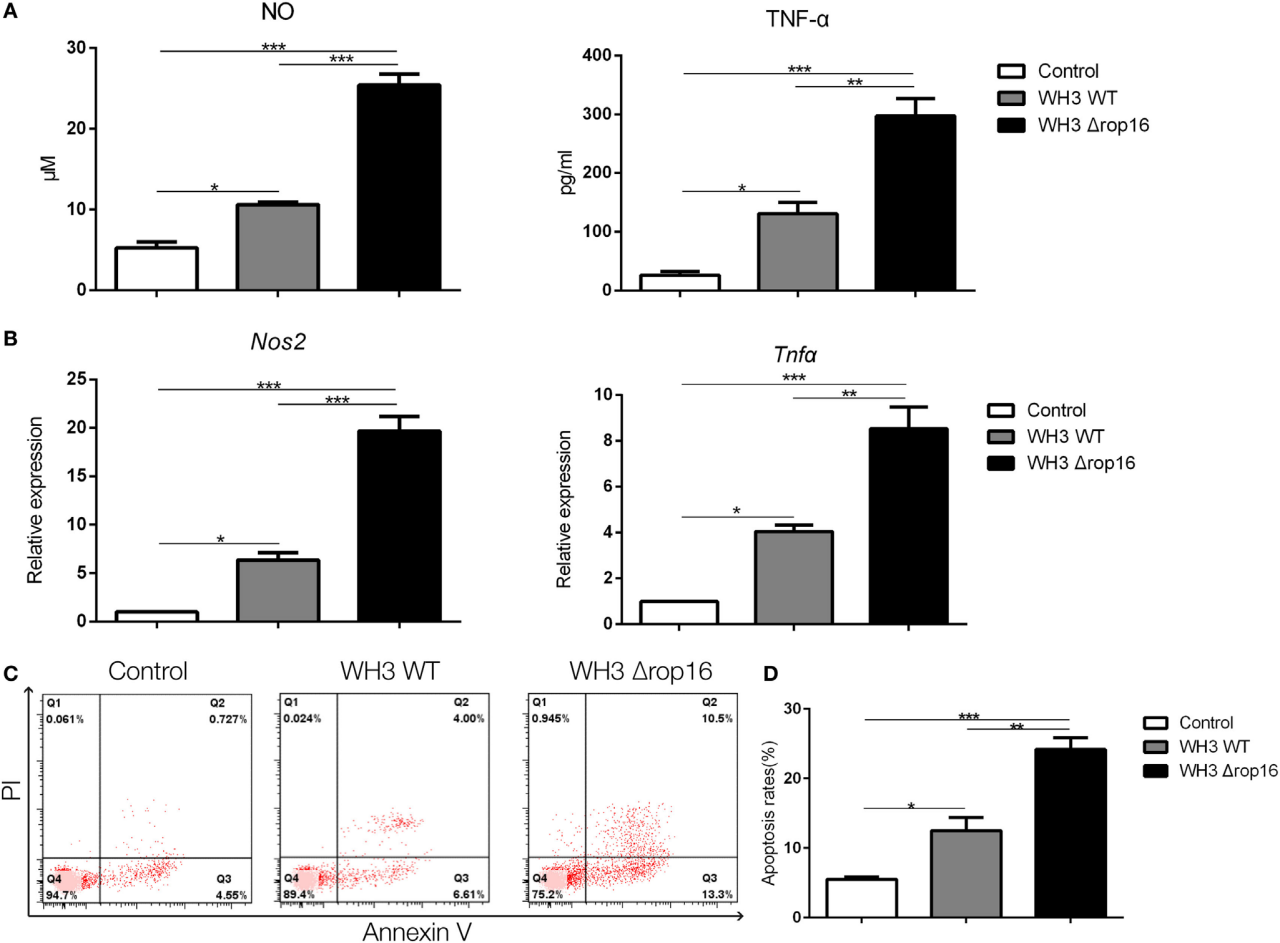
The high production of nitrite oxide (NO), inducible nitric synthase (iNOS), and tumor necrosis factor (TNF)-α and apoptosis of trophoblasts co-cultured with macrophages infected with the WH3Δ*rop16* tachyzoites. **(A)** The culture supernatants were collected and analyzed for NO and TNF-α production. **(B)** The relative mRNA expressions of iNOS and TNF-α after co-culture. **(C)** Increased apoptosis of trophoblasts co-cultured with WH3Δ*rop16* infected macrophages. **(D)** Total, early, and late apoptosis of placental trophoblasts (*p < 0.05, **p < 0.01, ***p < 0.001).

Compared to the WH3 WT group, macrophages infected with WH3Δ*rop16* strain produced a higher level of NO, iNOS, and TNF-α (Figures 2A,B). To elucidate the placental trophoblasts apoptosis, we detected cell apoptosis of placental tissues by fluorescein isothiocyanate/propidium iodide (FITC/PI) staining assay. Compared to the WH3 WT group, the number of total, early, and late apoptotic cells of trophoblasts was elevated when co-cultured with WH3Δ*rop16* strain-infected macrophages for 24 h (Figure 2C). The remarkable apoptosis of trophoblasts caused by WH3Δ*rop16* strain was observed (Figure 2D).

### WH3Δ*rop16* Strain Infection Induced Adverse Pregnancy

**Figure 3:**
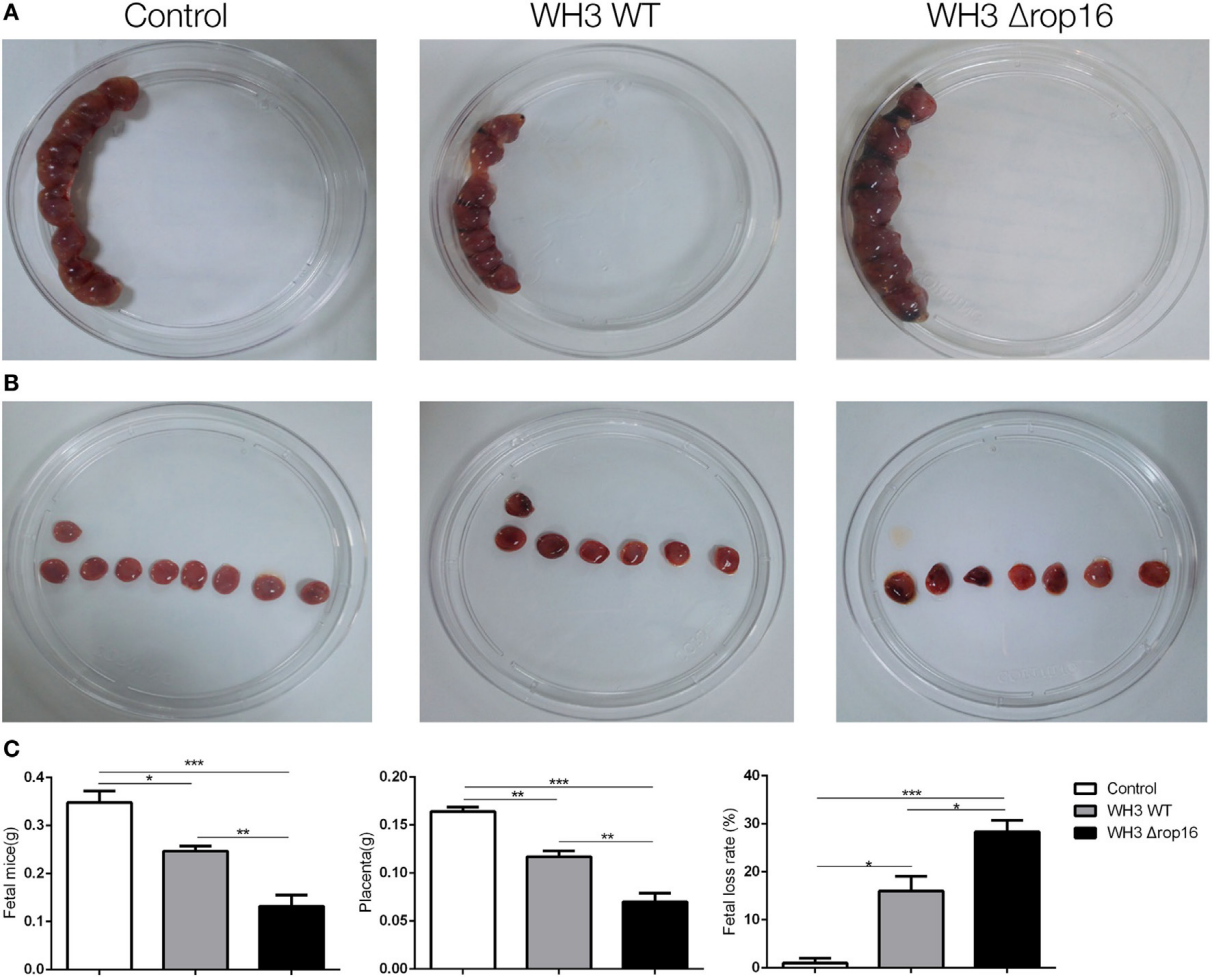
*Toxoplasma gondii* infection induced adverse pregnancy outcomes in mice. **(A,B)** The uteruses of WH3Δ*rop16*-infected mice presented notable fetal resorption and placental bleeding in comparison with the WH3 WT group. **(C)** The weights of fetuses, placentas, and rate of fetal loss were measured at day 6 post infection. The fetal loss rate was calculated by ratio of resorption sites to the total number of implantation sites. (*p < 0.05, **p < 0.01, ***p < 0.001).

Most samples of the pregnant uteruses showed significant fetal resorption and placental hemorrhage in the mice infected with the tachyzoites of WH3Δ*rop16* in comparison with the WH3 WT group (Figures 3A,B). The weights of fetuses, placentas, and rate of fetal loss were determined at day 6 post infection. The weight loss of fetuses and placentas was observed and the rate of fetal loss significantly increased in mice infected with WH3Δ*rop16* strain compared to WH3 WT strain (Figure 3C).

### WH3Δ*rop16* Strain Infection Induced Immune Bias to Th1 in the Spleens and Placentas of Pregnant Mice

**Figure 4:**
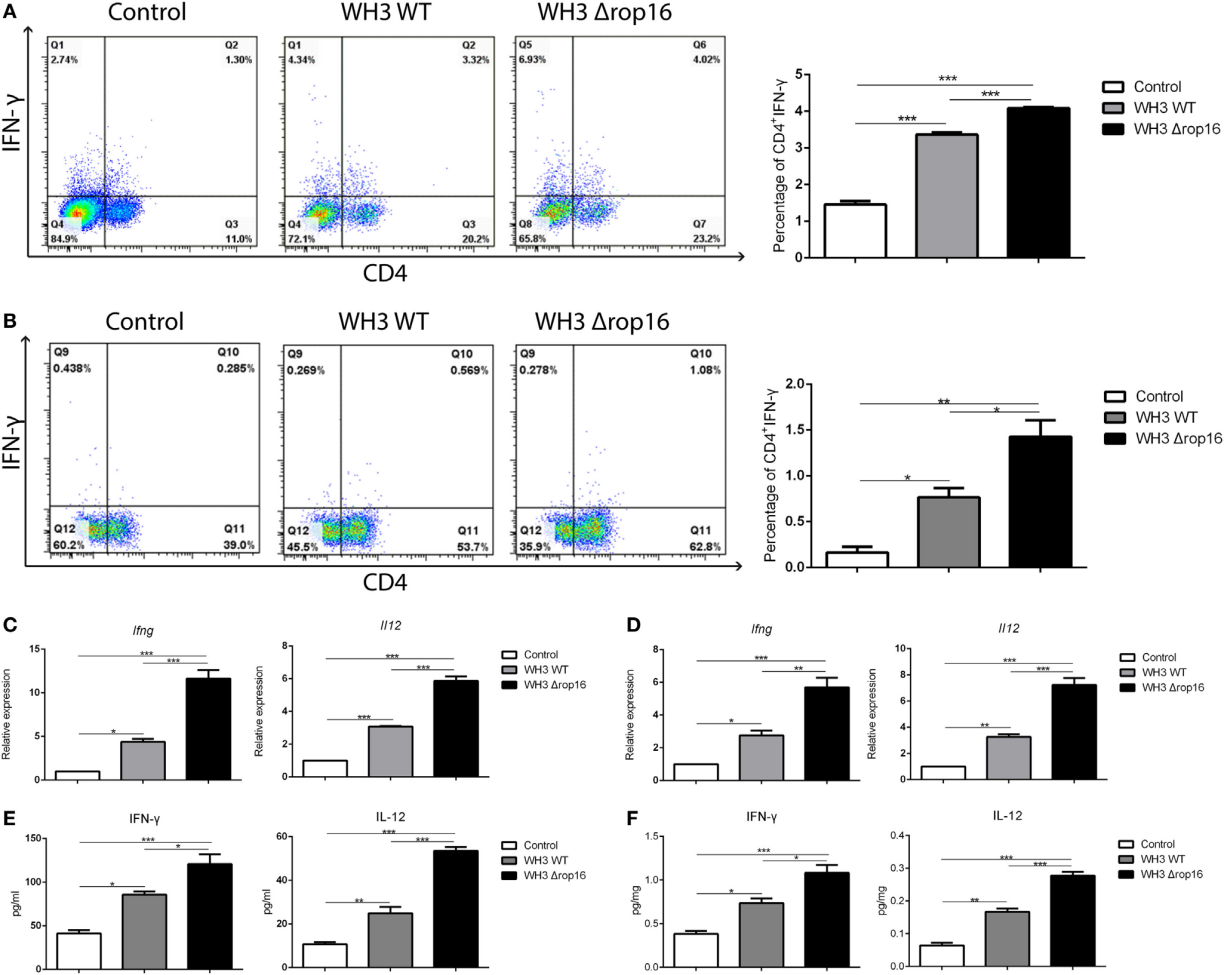
WH3Δ*rop16* strain infection upregulated Th1 response of pregnant mice. Increased percentage of Th1 (CD4^+^IFNγ^+^) cell population in the spleens **(A)** and placentas **(B)** of WH3Δ*rop16* tachyzoites compared to the control. The relative mRNA expressions of interferon (IFN)-γ and interleukin (IL)-12 were sharply increased in the spleens **(C)** of WH3Δ*rop16*-infected mice and placentas **(D)**. The supernatants were tested for IFN-γ and IL-12 in the spleens (pg/ml) **(E)** and placentas (pg/mg) **(F)** by enzyme-linked immunosorbent assay (*p < 0.05, **p < 0.01, ***p < 0.001).

We examined the IFN-γ in the lymphocytes of the spleen and placenta tissues using FCM to investigate the immune bias at maternal–fetal interface of the pregnant mice after *T. gondii* infection. The expression of IFN-γ and IL-12 were analyzed using qRT-PCR and ELISA in the spleens and placentas. Compared to WH3 WT group, Th1 subsets (CD4^+^IFNγ^+^) of WH3Δ*rop16* inoculated mice were remarkably elevated (Figures 4A,B). The data were further confirmed by using qRT-PCR and ELISA (Figures 4C–F). Consistent with the IFN-γ assay, the expression of IL-12 (Figures 4C–F) was synchronously increased in the mice of WH3Δ*rop16* infection.

### Th17 Response Was Upregulated in the Spleens and Placentas of the Pregnant Mice Infected With WH3Δ*rop16* Strain

**Figure 5:**
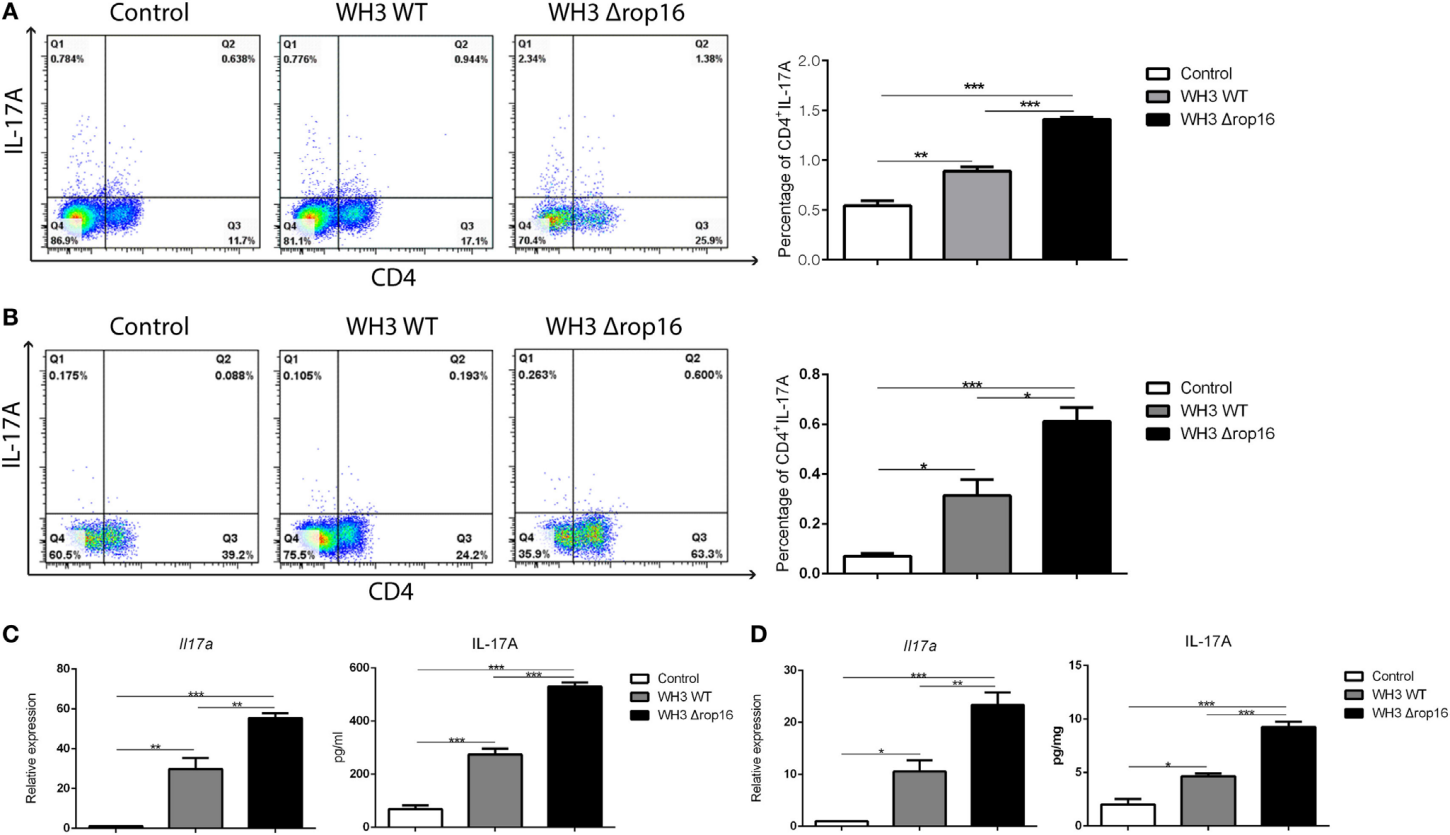
WH3Δ*rop16* strain infection upregulated Th17 response in the spleens and placentas of the pregnant mice. The mice infected with WH3Δ*rop16* strain expressed a high level of interleukin (IL)-17A in the spleens **(A)** and placentas **(B)** determined by flow cytometry. IL-17A mRNA expression in the animals infected with WH3Δ*rop16* in the spleens **(C)** and placentas **(D)**. The IL-17A in the supernatants of spleens (pg/ml) **(C)** and placentas (pg/mg) **(D)** confirmed by enzyme-linked immunosorbent assay (*p < 0.05,**p < 0.01,***p < 0.001).

We examined the Th17 cytokine (CD4^+^IL-17A^+^) in the spleens and placentas in the three groups of mice by FCM and found that the mice infected with WH3Δ*rop16* strain exhibited a high expression of IL-17A (Figures 5A,B). The level of IL-17A mRNA expression in WH3Δ*rop16* group was significantly increased when compared to WH3 WT (Figures 5C,D), which is in parallel with the result of IL-17A detection in supernatants by ELISA (Figures 5C,D).

### Percentage of Tregs Decreased in the Spleens and Placentas of the Pregnant Mice Following WH3Δ*rop16* Strain Infection

**Figure 6:**
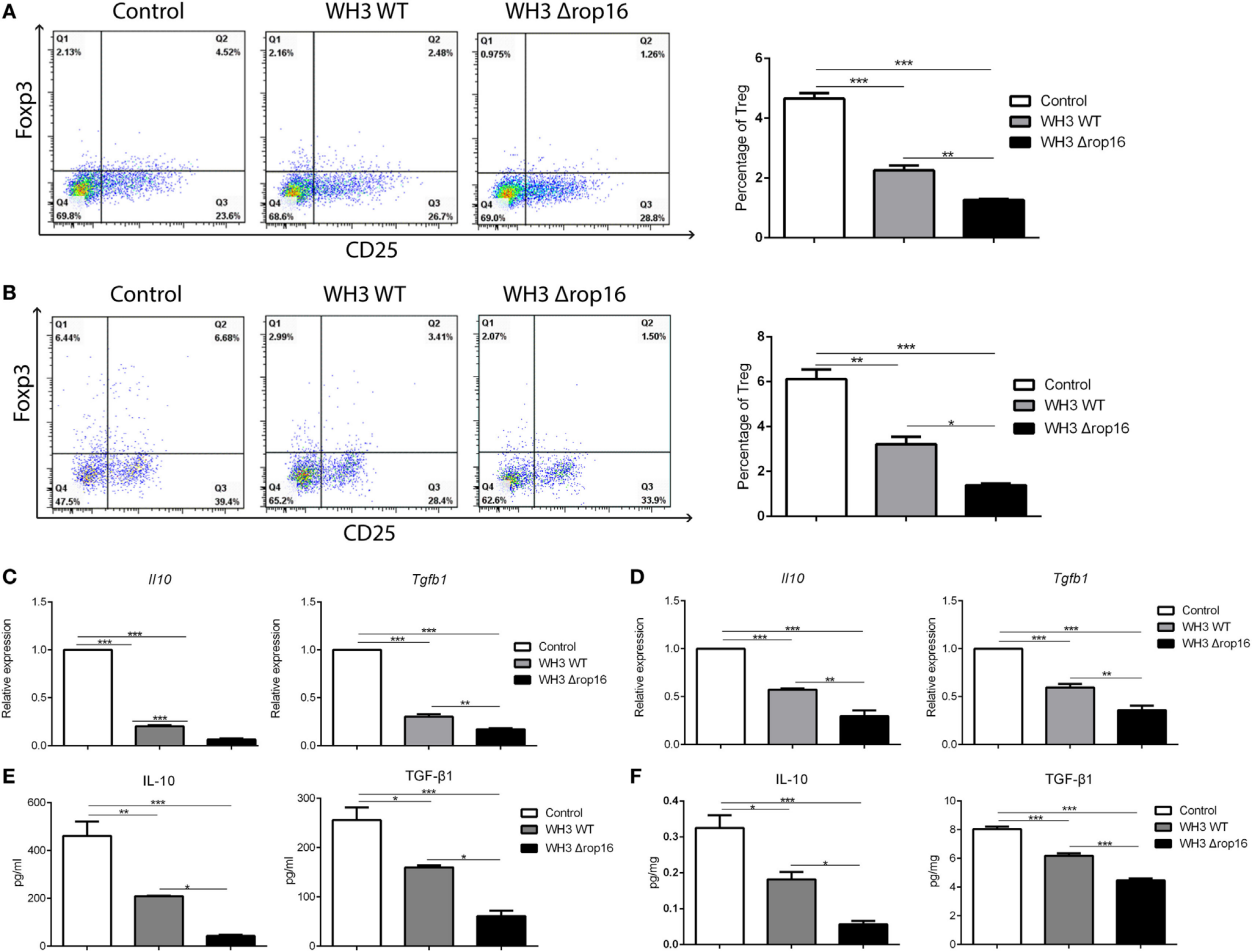
*Toxoplasma* WH3Δ*rop16* strain infection induced impairments of T regulatory cells (Tregs). Tregs cell population in the spleens **(A)** and placentas **(B)** were detected by flow cytometry. Tregs number was significantly decreased in WH3Δ*rop16* infected group compared to control group. The relative mRNA expressions interleukin (IL)-10 and transforming growth factor beta 1 (TGF-β1) in the spleens **(C)** and placentas **(D)** were markedly reduced in WH3Δ*rop16* group. The levels of IL-10 and TGF-β1 in the spleens (pg/ml) **(E)** and placentas (pg/mg) **(F)** were significantly decreased in the group of WH3Δ*rop16* (*p < 0.05, **p < 0.01, ***p < 0.001).

To clarify whether the mutant WH3Δ*rop16* strain infection can negatively affect the Tregs population, we detected the percentage of Tregs in the splenocytes and the cells isolated from the placentas of pregnant mice by FCM (Figures 6A,B). The results revealed that Tregs were significantly dampened in WH3Δ*rop16*-infected mice. Accordingly, relative mRNA expression of IL-10 and TGF-β1 in the spleens (Figure 6C) and placentas (Figure 6D) was synchronously reduced in WH3Δ*rop16* group compared to WH3 WT group. In addition, the levels of IL-10 and TGF-β1 in the spleens (Figure 6E) and placentas (Figure 6F) remarkably declined in the WH3Δ*rop16*-infected mice when compared to WH3 WT mice.

**Figure 7:**
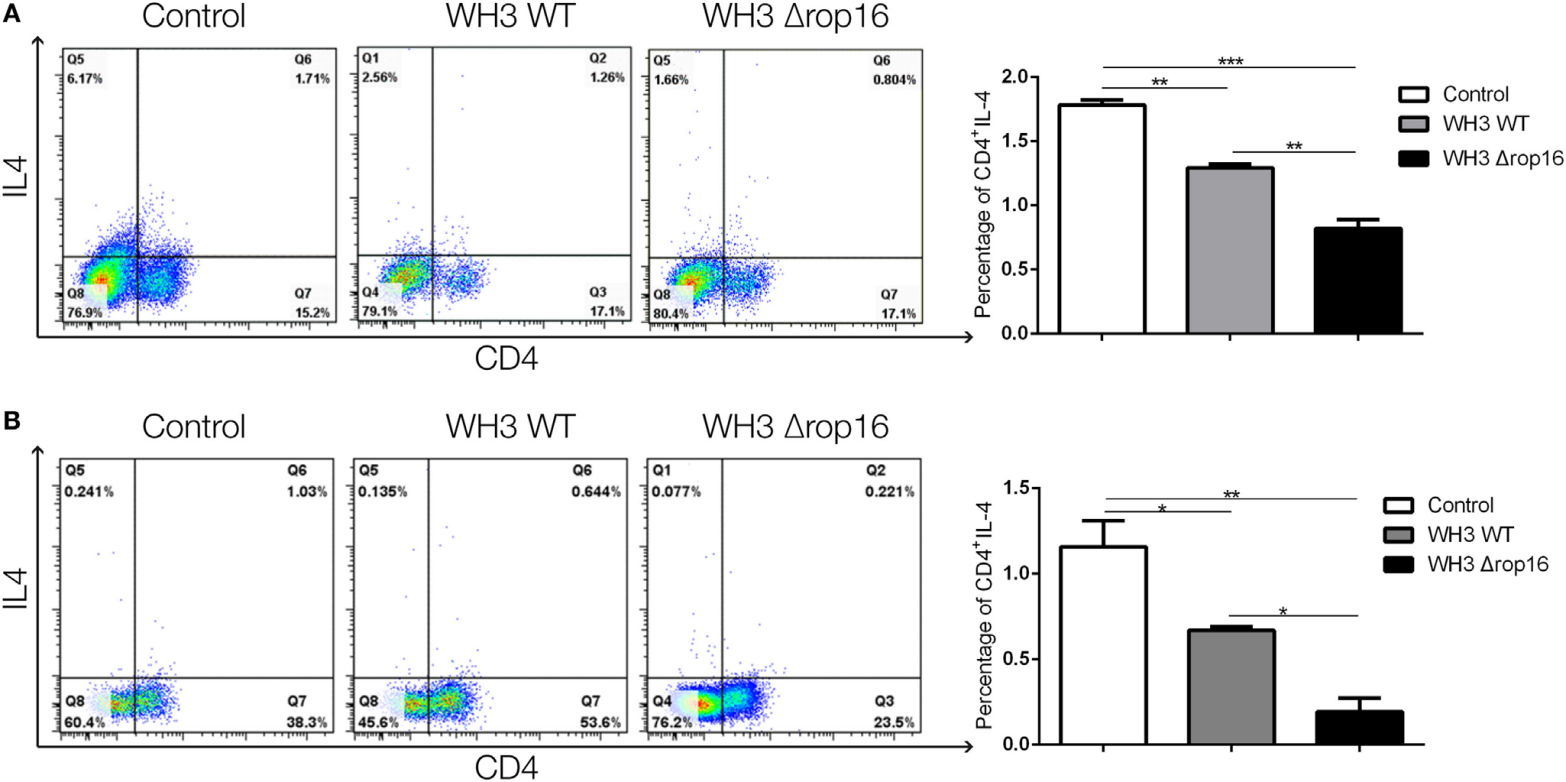
*Toxoplasma* WH3Δ*rop16* strain infection downregulated Th2 response of pregnant mice. The expression of IL-4 in WH3Δ*rop16* group in the spleens **(A)** and placentas **(B)** compared to the control group determined with flow cytometry (*p < 0.05, **p < 0.01, ***p < 0.001).

Compared to the WH3 WT-infected C57BL/6 pregnant mice, the animals inoculated with WH3Δ*rop16* tachyzoites presented a diminished expression of IL-4 determined by FCM (Figures 7A,B).

## Discussion

*Toxoplasma gondii* is one of the common causative agent of prenatal infections during pregnancy (15, 16). The parasite may be transmitted vertically by tachyzoites that are passed to the fetus *via* the placenta, leading to miscarriages, stillbirths, and other adverse pregnant outcomes depending on the stage of the pregnancy at which infection takes place (37). Most strains which infect humans in Europe are type II and a large proportion of cases of congenital toxoplasmosis are asymptomatic at birth if the mother acquired the infection after the second trimester of gestation (38). It has been well recognized that vertical transmission of the parasite, particularly type I virulent strain of *Toxoplasma* with superior migratory capacity, may directly cause abnormal pregnancy. However, the non-virulent strains such as PRU or ME49 may induce a decrease in the fertility if infection occurs during the later phase of gestation (39). Previous studies indicated that neither parasites nor their DNAs are detectable in the diseased tissue samples of some abnormal pregnancies and we attempted to examine the parasite by bioassay or its derived DNAs by PCR from placentas and tissues of aborted fetuses in the pregnant women, some of whom showing positive IgG antibodies against *Toxoplasma*, but no positive results were obtained (data not shown). Consistent results were also reported by Senegas in murine model (40). All of these data imply that the resorption, abortion, or fetal damage might be indirectly due to lesions of the placenta induced by maternal biased immunity such as Th1/IFN-γ polarized immune response (41) and that the maternal immunity-associated but not viable parasite-generated “sterile” pathology actually takes place in abnormal pregnant consequences caused by *Toxoplasma* infection. Herein, we focused on the maternal immunity associated rather than the mother-to-child vertical transmission causative adverse outcomes of pregnancy. Our results strongly suggest that the immunity subversion-related pathogenesis is involved in early gestation induced by *T. gondii* but not due to a direct invasion of the parasite in adverse pregnancies.

It has been reported that the consequences of congenital toxoplasmosis vary in genotypes of *Toxoplasma* strain (42). Strains of *T. gondii* from Europe and North America belong to three distinct clonal lineages (type I, type II, and type III) which differ phenotypically in virulence (43, 44). Recent studies revealed that polarization of alternatively activated macrophage (M2) or classically activated macrophage (M1) of host macrophages depends on the polymorphism of ROP16 or GRA15 of *Toxoplasma* at early stage of infection. One site mutation of ROP16 at 503L/S would determine the activity or inactivity of ROP16 in phosphorylation of Stat6/Stat3. Type I and type III strains carry ROP16_I/III_, which strongly drives M2 bias but ROP16_II_ is negligible (30, 45, 46), while type II strains infected macrophages are classically activated through bypassing TLRs and directly activating NF-κB by the dense granule protein GRA15_II_ (28, 30). Interestingly, we found that type Chinese 1 (ToxoDB #9) strains carry both ROP16_I/III_ and GRA15_II_ (33, 34). Thus, we postulated that Chinese 1 strains might have immunopathogenesis which is distinct from the archetypical strains circulating in the other parts of the world. Herewith, we constructed *T. gondii* WH3Δ*rop16* strain based on CRISPR/Cas9 strategy. The genetically manipulated parasite WH3 strain with *rop16_I/III_*-deficient and *gra15_II_*-dominant background (WH3Δ*rop16*/*gra15_II_*), which resembles the archetypical type II of European/North American strains, may be prone to induce adverse pregnant outcomes *via* the mechanism of subverting maternal immunotolerance. We noted that WH3Δ*rop16/gra15_II_* efficiently *in vitro* drove macrophage polarization toward M1 cells, producing NO, TNF-α, and iNOS, and gave rise to trophoblast cells apoptosis compared to WH3 WT strain. We also found that the WH3Δ*rop16*/*gra15_II_* strain induced characteristic Th1-biased and Th17-involved inflammatory response *in vivo* at maternal–fetal interface featured by high expression of IFN-γ and IL-12, and resulted in more severe adverse pregnant consequences in murine model. These results suggest that ToxoGRA15_II_ is one of the key factors involved in the imbalance of maternal immune tolerance during pregnancy which is associated with M1/Th1/Th17 skewed response. These results are in correspondence with the findings previously reported. Surely, in addition to GRA15_II_, other *Toxoplasma*-derived effectors of type Chinese 1 strain may also contribute to the pathology of immunity imbalance in pregnant animals.

In the construction of the plasmids, insertion of the pyrimethamine resistance gene (dihydrofolate reductase-thymidylate synthase, DHFR-TS*) has been frequently used as the selectable marker for transformant screening of CRISPR/Cas9-based genetic deletion of *Toxoplasma* (35, 47). The DHFR-TS* is not believed to evoke the detectable phenotypical alterations in *Toxoplasma*-infected host, since it is a modified endogenous gene that exists in wild-type strains of the parasite. So far, no evidence has shown its immunogenicity which is able to induce undesirable immune response in its host cells. Moreover, we here did not use ROP16_II_ insertion substituting ROP16_I/III_ because, as stated above, ROP16_I/III_, rather than ROP16_II_, decides ROP16 kinase activity on Stat6/Stat3 and induces M2 polarization which is pivotal to M2 and subsequent Th2 response (45, 46). We deleted ROP16_I/III_ in order to generate a GRA15_II_ dominant transformant to explore the pathology of adverse pregnancy and the mechanisms of pathogenesis caused by the atypical strain of Chinese 1 epidemic in China.

Despite of expressing paternal antigens, the human allogenic fetus is histocompatible with the maternal immune system, presenting an immune down-modulation on CD8^+^ T cells, Th1, and Th17 cells, which contributes to creation of an immune-privileged environment at the maternal–fetal interface. Previous investigations indicated that microbial endotoxin (LPS) administration to pregnant mice prior to delivery (16.5 day post coitum) causes a Tregs and Th17 cells involved imbalance at the maternal–fetal interface and in the spleen, inducing preterm labor (48). However, we found that mice infected with the WH3Δ*rop16/gra15II* strain at the early stage (seventh day of fertility) presented remarkable manifestations of adverse pregnant results, indicating that the mutant strain infection of *Toxoplasma* may lead to abnormal pregnant outcomes in which Tregs and Th17 cells are also involved. Various types of immune cells, such as Tregs, play a pivotal role in the maintenance of normal gestation (11, 49–52). Our study revealed that the percentage of Tregs and expression of IL-10 and TGF-β1 by Tregs as well as M2 cells were significantly diminished in the spleen and placenta tissues of early phase and metaphase of pregnancy of mice infected with the WH3Δ*rop16*/*gra15_II_* strain, which is consistent with the previous reports of low expression of IL-10 and TGF-β1 cytokines in mice with a high incidence of fetal rejection (53). This result demonstrates that the impairment of Tregs takes place in *T. gondii*-infected pregnant mice, particularly in mice infected with the strain of WH3 Δ*rop16*/*gra15_II_*.

A growing body of evidence indicates that Th17 cells are involved in infiltrative inflammation in patients with recurrent spontaneous abortions (54). The imbalance of Tregs/Th17 has also been seen in human abortions (54, 55). Our previous work revealed that *T. gondii* type II strain-derived molecule of ToxoGRA15_II_ is responsible for inducing M1 polarization of RAW264.7 cells *via* NF-κB activation (29, 56) eliciting host innate immunity and Th1-dominant and Th17-involved inflammatory response, and adverse pregnancy outcomes in mice (data in manuscript). Here, we also noted that expression of Th1 cytokines and IL-17A in splenocytes and placenta tissues was observably elevated in the pregnant animals infected with WH3Δ*rop16*/*gra15_II_* strain of type Chinese 1 (Figures 4 and 5), implying a direct involvement of increased Th17 cells in fetal loss. Additional studies are on going to explore the IFN-γ/NK-involved mechanism at the maternal–fetal interface in abnormal pregnancies associated with *T. gondii* infection.

In summary, our data demonstrate that WH3Δ*rop16* strain with GRA15_II_ background of *T. gondii* type Chinese 1 may cause subversion of immune tolerance at the maternal–fetus interface and in systemic immunity, leading to adverse pregnancy outcomes, which is associated with the Th1 and Th17 biased response. This study would provide an explanation for pregnancy failure caused by non/less virulent strains of type II and offer a deep insight into the pathogenesis of abnormal pregnancy caused by strains of *T. gondii* type Chinese 1 dominating in China.

## Ethics Statement

The mice were treated in compliance with the Chinese National Institute of Health Guide for the Care and Use of Laboratory Animals. All procedures were followed strictly according to the ethical standards formulated by Institutional Review Board of Anhui Medical University Institute of Biomedicine (permit no: AMU26-081108).

## Author Contributions

JS and CW conceived and designed the experiments. CW, WC, QY, and TX performed the experiments. WC, CW, and JS drafted the manuscript. All authors contributed to discussion of the results followed by writing and reviewing the manuscript.

## Conflict of Interest Statement

The authors declare that the research was conducted in the absence of any commercial or financial relationships that could be construed as a potential conflict of interest.
